# Supplementary material for: Lineage specific transcription factor waves reprogram neuroblastoma from self-renewal to differentiation
Source: Nat Commun. 2024 Apr 23;15:3432. doi: 10.1038/s41467-024-47166-y (PMC11039666; doi:10.1038/s41467-024-47166-y)

Supplementary Figures for “Lineage specific transcription factor waves reprogram neuroblastoma from self-renewal to differentiation”

Supplementary Figure 1

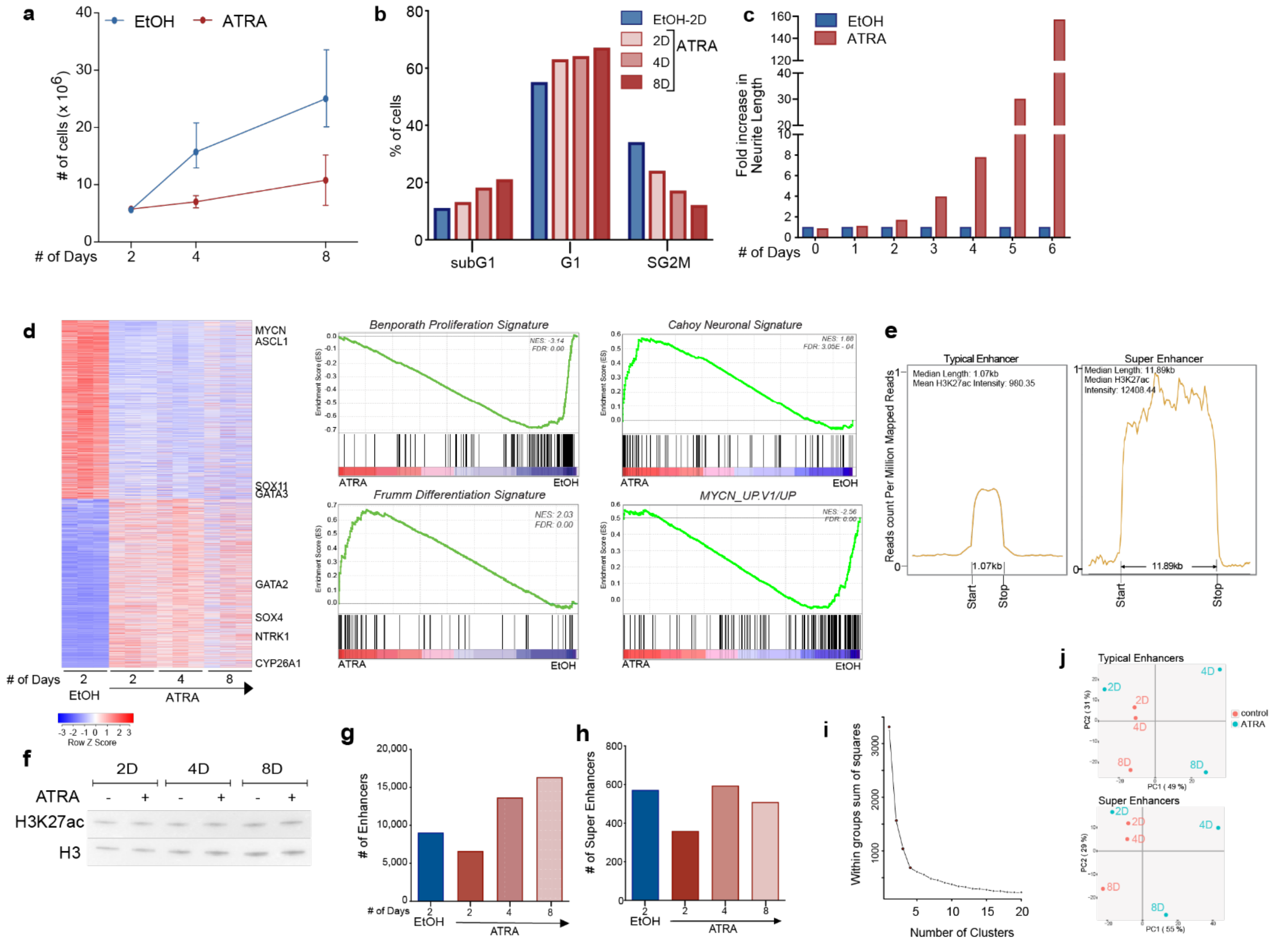

Fig. S1 (Extension of Fig. 1): ATRA treatment leads to growth arrest and differentiation and changes in SE landscape of Neuroblastoma cells

a. ATRA treatment leads to 50% decrease in cell number by 4 days and 90% by 8 days. Bars show the mean, with error bars representing the standard deviation.
b. ATRA induced growth arrest as measured by 20% increase in cells in G1 phase and 20% decrease in cells in G2/M phase by 8 days.
c. ATRA mediated differentiation was measured by 8-fold increase in neurite length by 4 days.
d. Left Panel: Heat map showing significant (FDR ≤ 0.01) differentially expressed genes after ATRA treatment. Right Panel: Gene set Enrichment analysis (GSEA) shows negative regulation of Benporath Proliferation and MYCN UP.V1/UP gene sets and positive enrichment of Frumm Differentiation and Cahoy neuronal gene set following ATRA treatment.
e. Metagene representations of the mean ChIP-seq signal across all typical enhancers and super-enhancer domains, identified in the self-renewing state. Metagenes are centered on the enhancer region, and the length of the enhancer reflects the difference in median lengths (1.07kb for typical enhancers, 11.9kb for super-enhancers). Additional 5 kb flanking each enhancer region is also shown.

f. Western blow analysis showing total levels of H3K27ac untreated or with 2, 4, and 8 days of ATRA treatment. H3 was used as loading control.
g. Total number of typical enhancers identified using H3K27ac Chip-seq under untreated or with 2, 4, and 8 days of ATRA treatment.
h. Total number of super-enhancers identified using H3K27ac Chip-seq under untreated or with 2, 4, and 8 days of ATRA treatment.
i. Elbow chart showing the SSE (sum of squared errors) after running k-means clustering for k going from 1 to 20. A clear elbow is seen at k = 4, indicating that 4 is the best number of clusters.
j. Principle Component Analysis of typical (top) and super enhancer H3K27ac signal across ATRA treatment in KCNR cells.

Supplementary Figure 2

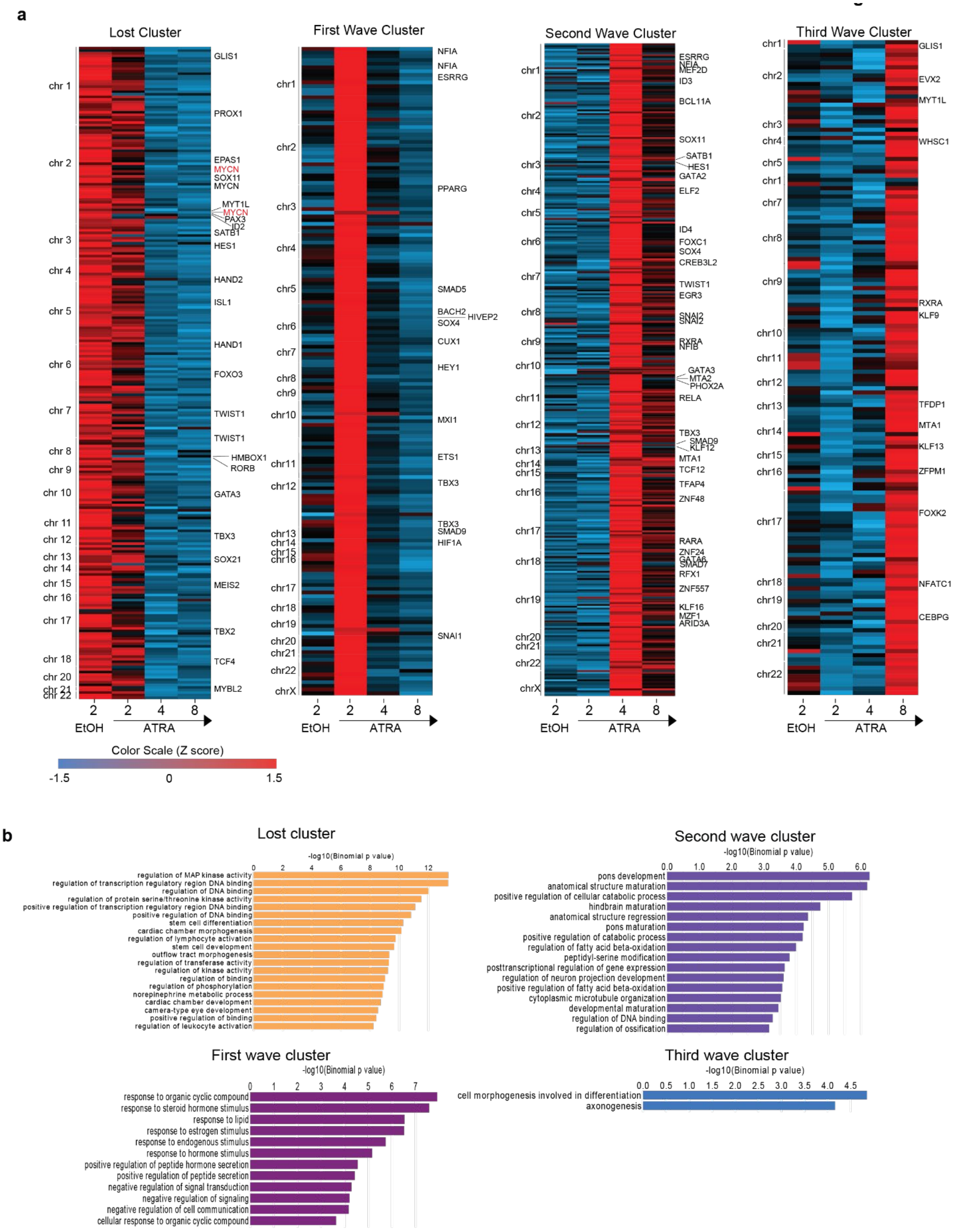

Fig. S2 (Extension of Fig. 1):

a. Heatmap showing H3K27ac signals of all SEs for the four clusters under untreated or with 2, 4, and 8 days of ATRA treatment.
b. Gene ontology terms associated with genes found using GREAT analysis (great.stanford.edu) on H3K27ac constituent peaks within SEs in each cluster.

Supplementary Figure 3

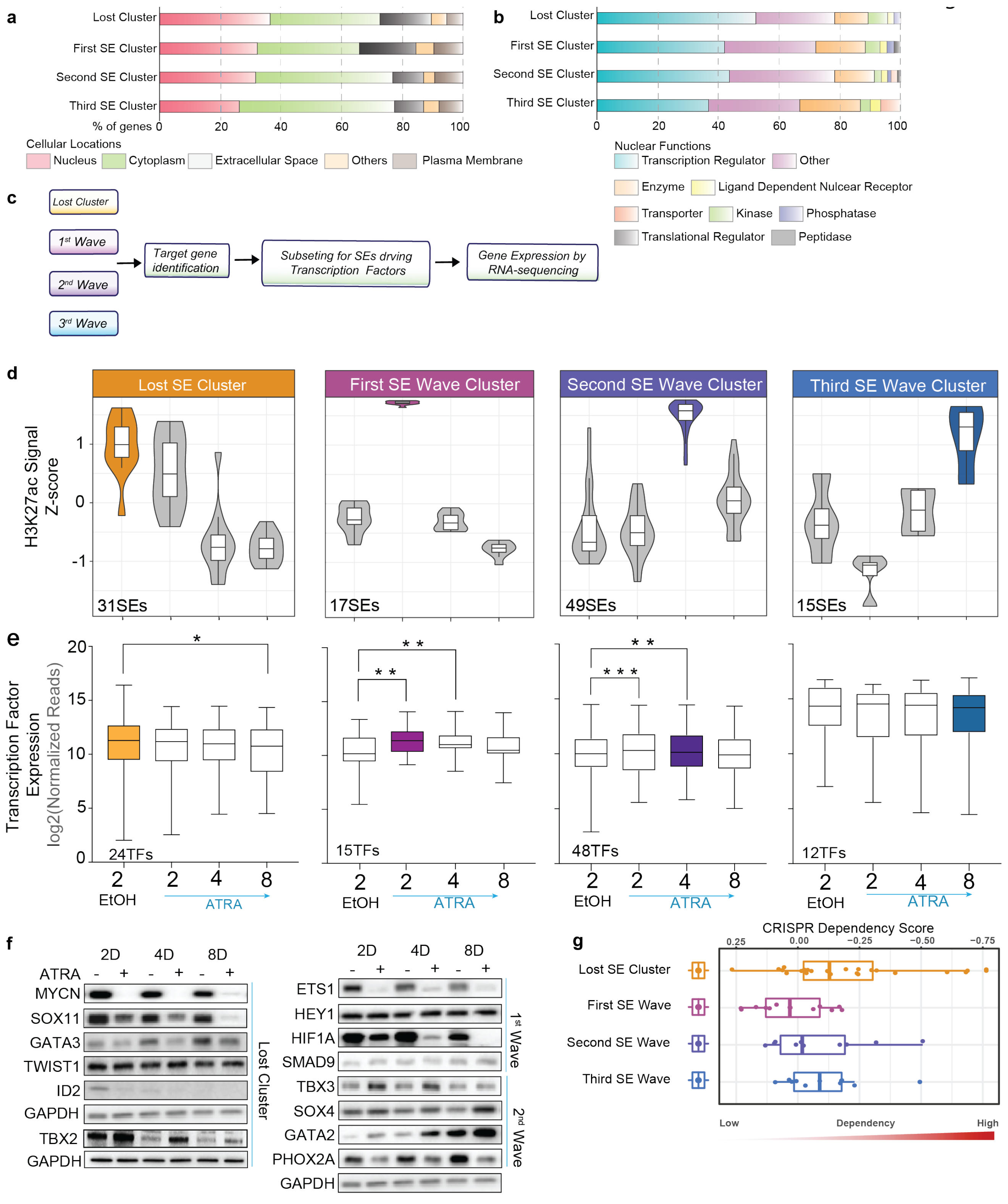

Fig. S3 (Extension of Fig. 2): SE driven Transcription Factors drive proliferation and differentiation of KCNR cells.

a. Stacked bar-chart showing the predicted cellular localization of the genes driven by the SEs in each cluster.
b. Stacked bar-chart showing the function of the SE cluster genes that are localized in the nucleus.
c. A schematic representation of the analysis steps following SEs identification in each cluster.
d. Violin plots of z-score of H3K27ac signal of SEs in each cluster. The lost cluster consists of 31SEs, the first wave cluster has 17SEs, the second wave cluster consists of 49 SEs, and the third cluster has 15SEs.
e. Bar graph showing expression of the TFs driven by the SEs in each cluster. The 31SEs in the lost cluster drives 24TFs where the loss in SE signal lead to significant decrease in the expression of the corresponding TFs after 8 days of ATRA treatment. Similarly gain in SE signal after 2 days and 4 days of ATRA treatment lead to significant gain in the expression of the TFs. However, no change in expression of SE driven TFs was observed for the third cluster. Boxplots show the median with quartiles, whiskers show the 1.5 × interquartile range in the data.
f. Western blot analysis of selected TFs of 3 clusters after 2,4 and 8 days of vehicle or ATRA treatment.
g. Box plots showing dependency of neuroblastoma cell line on all the TFs in each cluster for cell growth and proliferation. NB cells are most preferentially dependent on lost cluster TFs for growth and proliferation compared to TFs in other clusters. Data mined from Project Achilles genome wide CRISPR-Cas9 screen. Boxplots show the median with quartiles, whiskers show the 1.5 × interquartile range in the data

Supplementary Figure 4

a

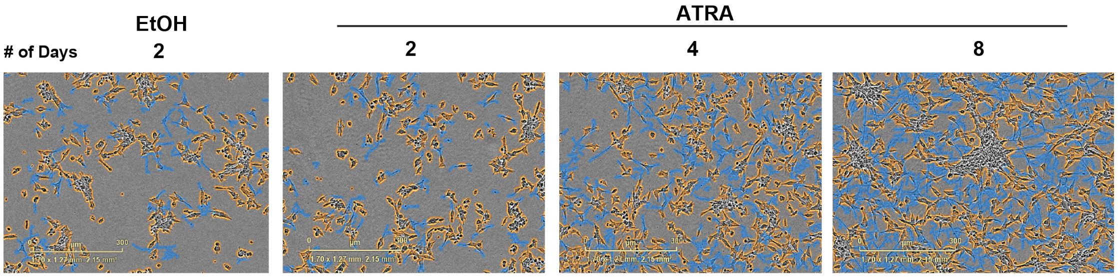

b

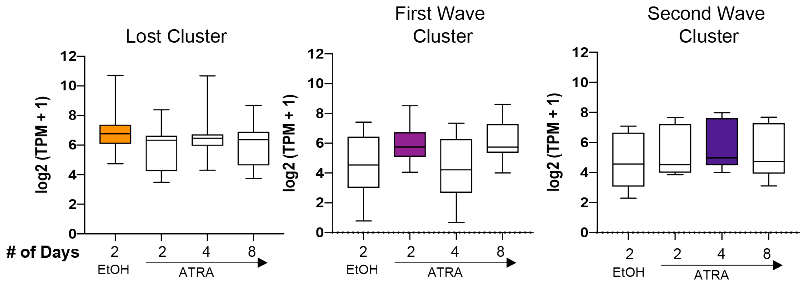

c TFs validated in LAN5

Lost	First Wave	Second Wave
TWIST1	SOX4	ZNF536
GATA3	SNAI1	ZNF48
ID2	SMAD9	TSHZ3
MYCN	SMAD5	TBX3
RORB	PPARG	SOX4
SOX11	NFIA	RELA
TBX2	HIF1A	PHOX2A
	HEY1	NFIA
	ETS1	KLF12
		GATA2

Fig. S4 (Extension of Fig. 2): ATRA treatment of LAN5 cells drives differentiation and regulates expression of TFs.

a. Representative images of LAN5 cells showing time dependent effect of 5μM ATRA treatment on cell morphology. The cell body is highlighted in yellow and the neurites are highlighted in blue.
b. Bar graph showing expression of the TFs driven by the SEs in each cluster (common with KCNR analyzed in Fig. 2C). Significant decrease in the expression of the TFs in lost cluster was observed 2, 4 and 8 days of ATRA treatment in LAN5 cells. Similarly, significant gain in expression of the TFs in first and second cluster was also observed in LAN5 cells after ATRA treatment. Boxplots show the median with quartiles, whiskers show the 1.5 × interquartile range in the data
c. List of the transcription factors identified in KCNR and validated in LAN5.

Supplementary Figure 5

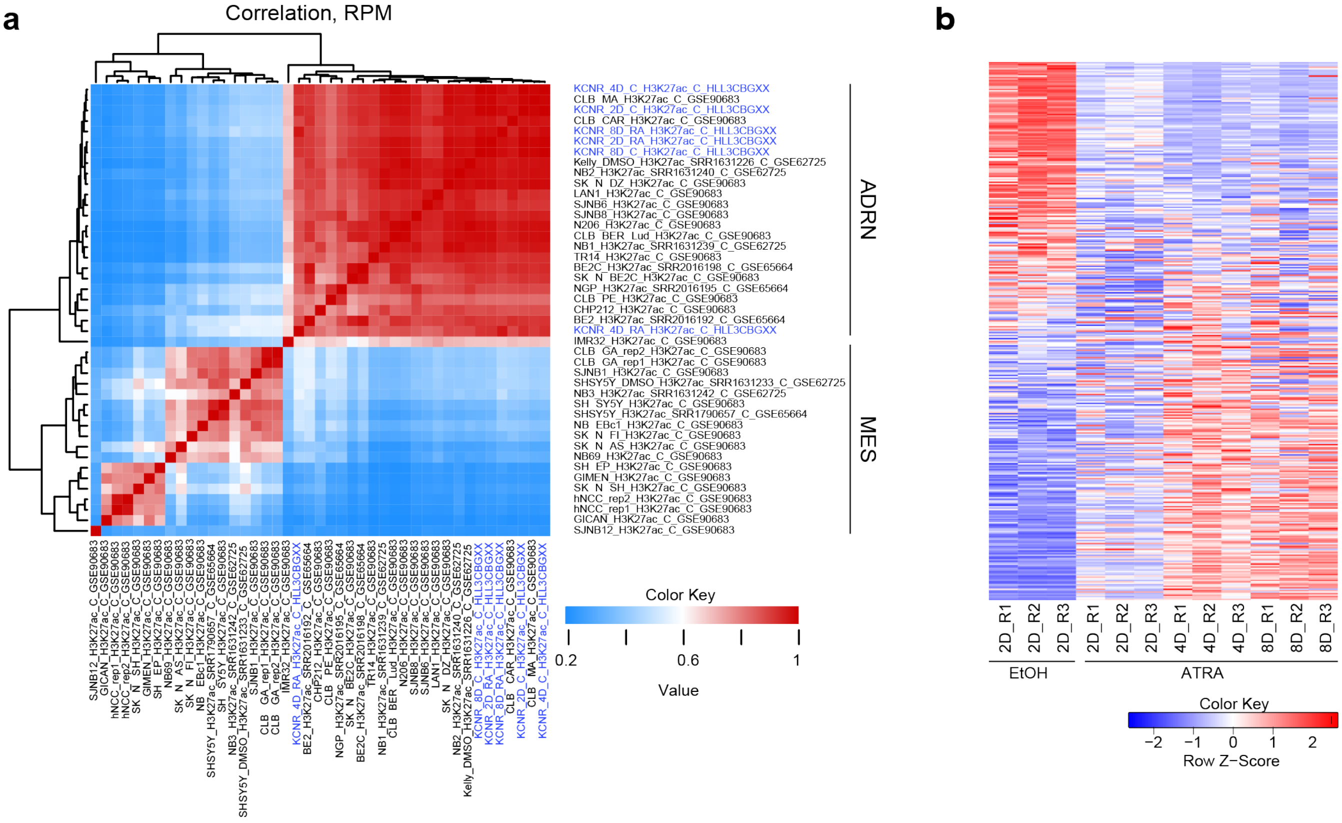

Fig. S5 (Extension of Fig. 3): KCNR cells are enriched in adrenergic cell signature

a. KCNR cells under both EtOH (C) and ATRA (RA) treatments cluster with other NB cells with an ADRN phenotype. ADRN: Adrenergic, MES: Meschenchymal.
b. Heatmap showing changes in ADRN gene signature following 2, 4, and 8 days of ATRA treatment.

Supplementary Figure 6

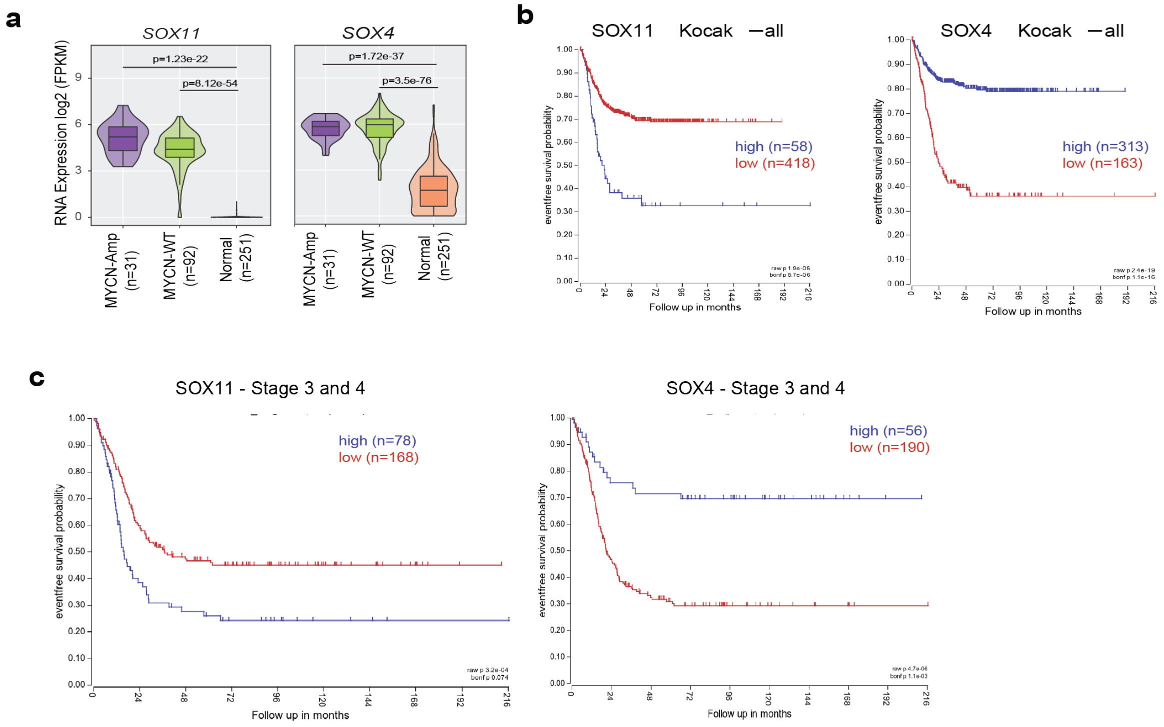

Fig. S6 (Extension of Fig. 3): SOX11 and SOX4 are expressed in NB cells and associated with prognosis in NB Tumors

a. Violin plots showing gene expression of SOX11 and SOX4 in MYCN-Amp neuroblastoma tumors, MYCN-WT neuroblastoma tumors, and normal tissues. Boxplots overlaid show the median with quartiles, whiskers show the 1.5 × interquartile range in the data.
b. Kaplan-Meier plots based on the expression of SOX11 (left panel) and SOX4 (right panel) in tumors from NB patients at all stages (R2 database: Kocak dataset).
c. Kaplan-Meier plots based on the expression of SOX11 (left panel) and SOX4 (right panel) in tumors from NB patients at stages 3 and 4 (R2 database: Kocak dataset).

Supplementary Figure 7

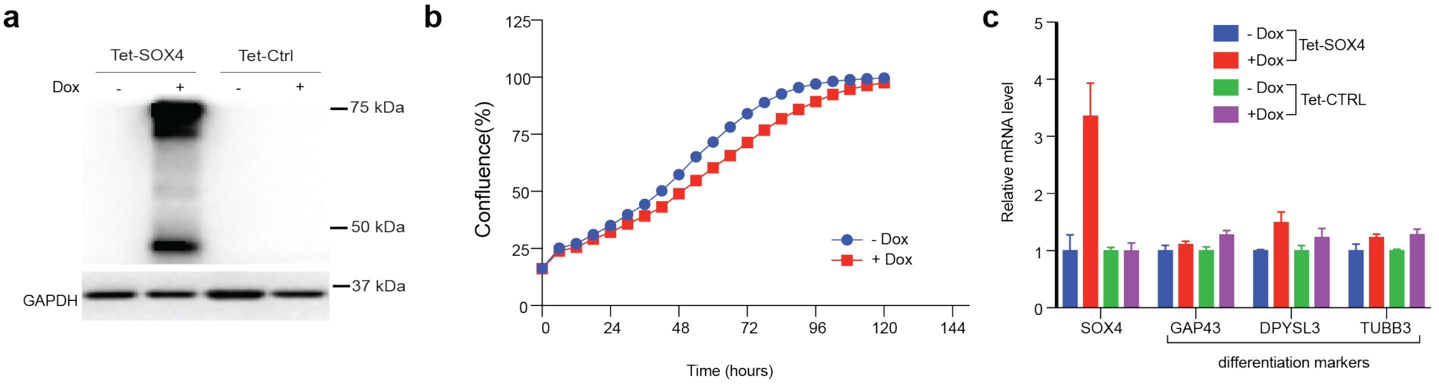

Fig. S7 (Extension of Fig. 5): Effect of SOX4 overexpression in KCNR cells

a. Western blot showing overexpression of Tet-inducible SOX4 expression in KCNR cell line.
b. Line graph showing no significant change in cell growth in KCNR cells overexpressing SOX4 compared to control cells.
c. Bar graph showing increase in SOX4 mRNA expression in SOX4 overexpressed cells compared to controls. Modest changes were observed in GAP43, DPYSL3 and TUBB3 expression upon increase in SOX4 expression. Data are shown with bar = average, error bars = standard deviation.

Supplementary Figure 8:

Figure S8

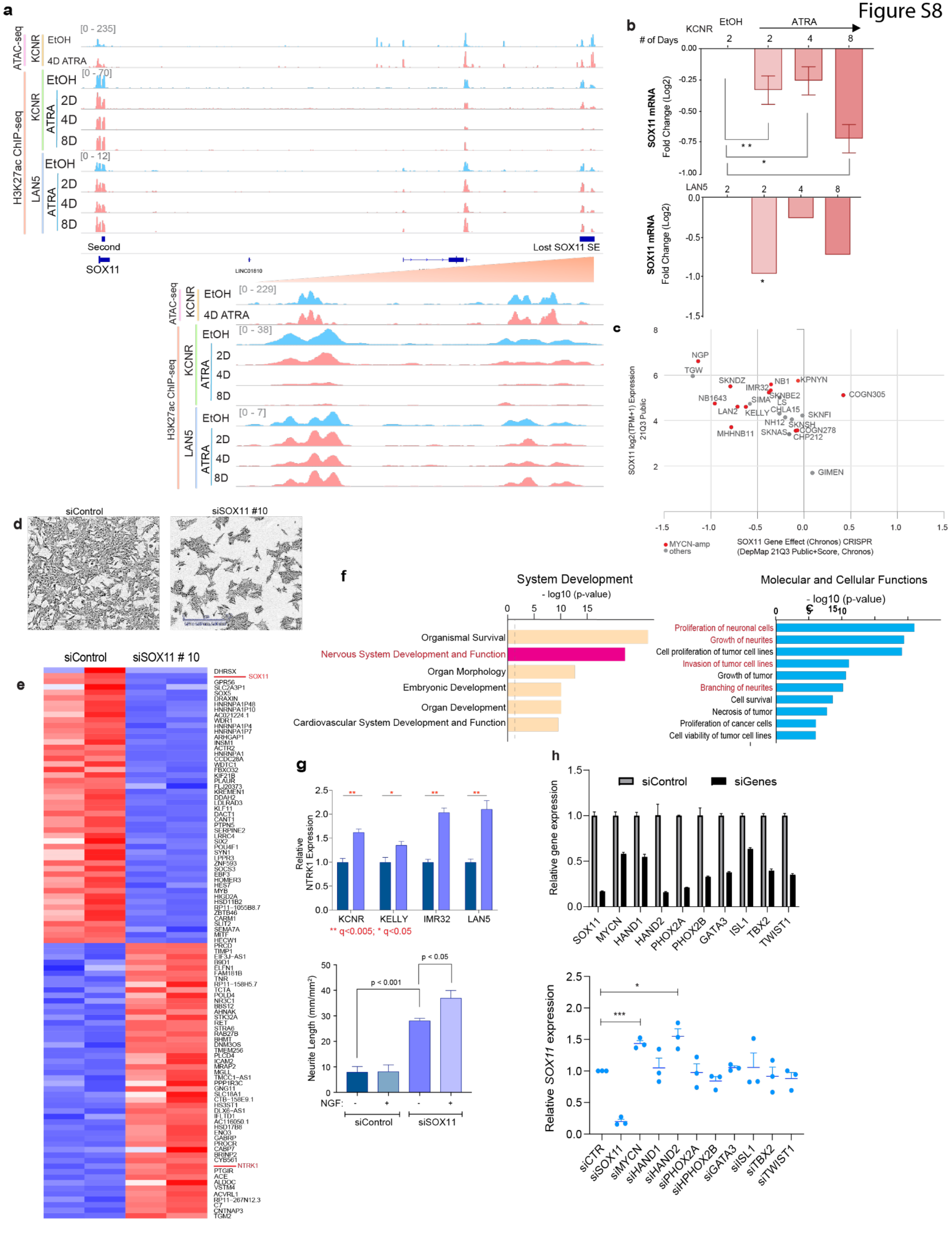

Fig. S8 (Extension of Fig. 6 and 7): SOX11 expression is regulated by SEs in NB cells

a. Representative ChIP-Seq tracks for H3K27ac in EtOH (blue) and ATRA treated cells (pink), showing loss of H3K27ac peaks at SOX11 SE in LAN5 cells. ChIP-seq read densities (y axis) are normalized and reported as reference-adjusted reads per million mapped reads (RRPM) in each sample. Super-enhancers are noted as purple bars.
b. Expression of SOX11 as determined by RNA-seq in KCNR and LAN5 cells in the presence and absence of ATRA. Log 0.75-fold increase in SOX11 expression was observed post ATRA treatment in both cell lines. Data are means ± SD for three replicates; P value generated from unpaired t test with Welch's correction.
c. Dot plot showing the dependency of individual NB cell line on SOX11 for growth and proliferation. Data mined from Project Achilles genome wide CRISPR-Cas9 screen.
d. Representative images of cells post SOX11 knockdown.
e. Heatmap showing top 50 upregulated and 50 downregulated genes ranked by statistical significance 48hrs post SOX11 inhibition in KCNR cells. Data are presented as normalized expression values of two biological replicates based on DESeq2 software analysis and p-adj <0.001. The color key represents the normalized expression values: blue (low) to red (high).
f. Most significant system development pathways regulated by SOX11 target genes (upper panel). The top molecular functions decreased by SOX11 silencing (lower panel) as defined by IPA based on DESeq2 differential gene expression analysis.
g. RT-qPCR analysis showing significant increase in NTRK1 relative mRNA levels after SOX11 silencing in NB cells (top) and increase in neurite length (bottom). Bars show mean +/- standard deviation. P values were generated from unpaired t test with Welch's correction.
h. RT-qPCR analysis showing decrease in gene expression after silencing of respective TFs in NB cells: for *SOX11* (top), where bars show mean +/- standard deviation, and other TFs (bottom), where center line indicates the mean and the error bars show the standard deviation.

Supplementary Figure 9 (Extension of Fig 7):

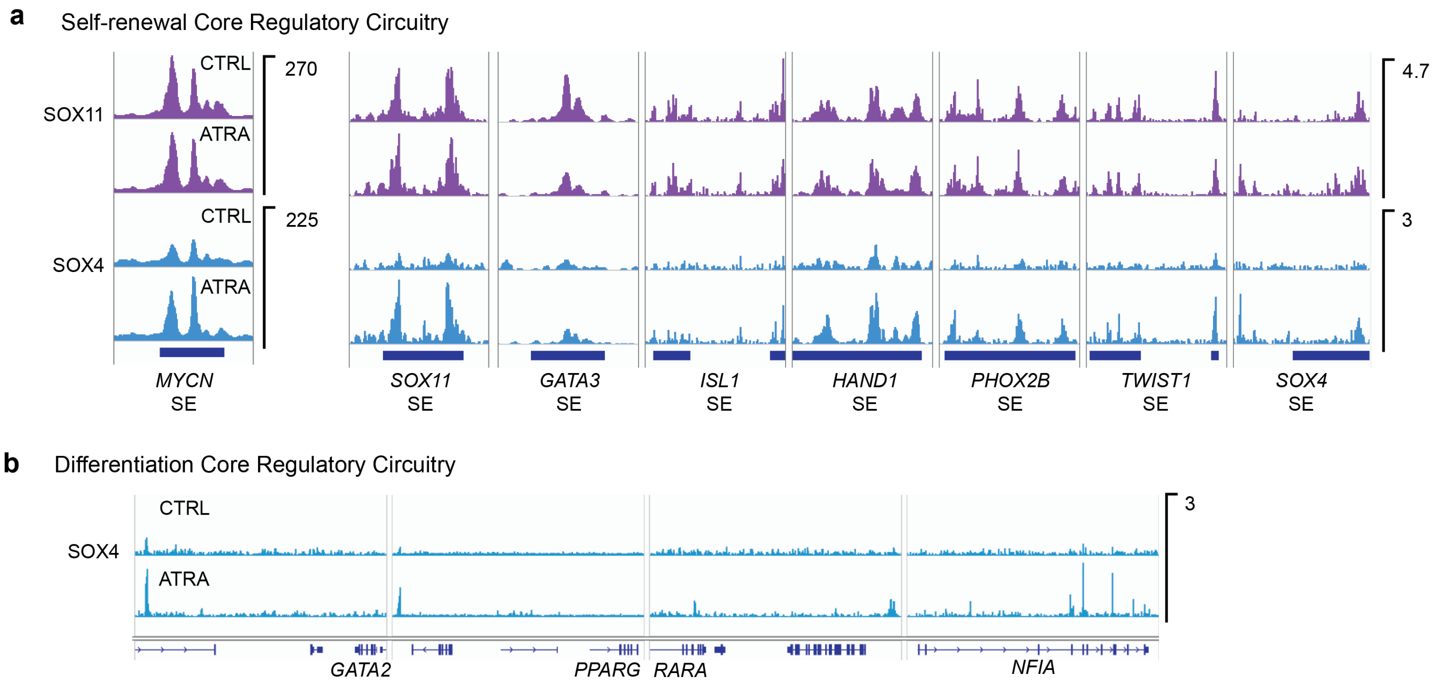

Fig. S9: Cut & Run assay showing binding of SOX11 and SOX4 on CRC TFs in KCNR cells

a. Tracks showing binding of SOX11 and SOX4 at multiple self-renewal CRC loci under both Ethanol and ATRA treated state.

b. Tracks showing binding of SOX4 at multiple differentiation CRC genes under both Ethanol and ATRA treated state.

Suppl. Table 1: sgRNA sequences for silencing SOX11 and SOX4 super enhancers

Name	sgRNA Sequence (5' - 3')
sgSOX11_SE.1-F	CACCGCCAAGTGAAACATCTGCAAC
sgSOX11_SE.2-F	CACCGACCCAGTCATTTCCTCCAAC
sgSOX11_SE.3-F	CACCGGGATCTGTCTATTTTCAACT
sgSOX11_SE.1-R	AAACGTTGCAGATGTTTCACTTGGC
sgSOX11_SE.2-R	AAACGTTGGAGGAAATGACTGGGTC
sgSOX11_SE.3-R	AAACAGTTGAAAATAGACAGATCCC
sgSOX4_SE.1-F	CACCGCAGTGCCGGGTAACAAAAGC
sgSOX4_SE.3-F	CACCGAACATAGCATGTGTACTTTG
sgSOX4_SE.1-R	AAACGCTTTTGTTACCCGGCACTGC
sgSOX4_SE.3-R	AAACCAAAGTACACATGCTATGTTC

Suppl. Table 2: siRNA sequences

siRNA	Company	Cat.
siControl	Qaigen	1027281
siMYCN	Qaigen	SI00076307
siHAND2	Qaigen	SI00131915
siPHOX2B	Qaigen	SI04202751
sicontrol	GE Dharmacon	D-001810-01-05
siTBX2	GE Dharmacon	L-012196-00-0005
siTWIST1	GE Dharmacon	L-006434-00-0005
siHAND1	GE Dharmacon	L-009812-00-0005
siPHOX2A	GE Dharmacon	L-012017-00-0005
siISL1	GE Dharmacon	L-011707-00-0005
siGATA3	GE Dharmacon	L-003781-00-0005
siSOX11	GE Dharmacon	L-017377-01-0005
siSOX4	GE Dharmacon	L-011779-00-0005

Suppl. Table 3: SOX11 siRNA sequences

Gene Name	GE Catalog #	Text Reference #	Sequence (5' -3')
SOX11	J-017377-09	#09	CAAGUAUGUUGGUACGUUA
	J-017377-10	#10	GAUAAGAUGUCGUGACGCA
	J-017377-11	#11	CCUCUAGGCUCCUCGAAGA
	J-017377-12	#12	GUUUGAAGCUUGUCGGUCU

Suppl. Table 4: qPCR primers

Primer Name	Sequence (5' - 3')
MYCN_F	GAGGACACCCTGAGCGATTC
MYCN_R	CTTGGGACGCACAGTGATGG
SOX11_F	GTCCAAGATCGAACGCAGGA
SOX11_R	CTTCTCGCTGTCCTTCAGCA
SOX4_F	GAAGCTGCTCAAAGACAGCG
SOX4_R	CTGGGCCGGTACTTGTAGTC
TWIST1_F	GCCGGAGACCTAGATGTCATT
TWIST1_R	TTTAGTTATCCAGCTCCAGAGTC
PHOX2A_F	CTCGGCTCCTCCAACTGC
PHOX2A_R	TTGTAGGGCACTGCCGAGTA
PHOX2B_F	CGCCGCAGTTCCTTACAAAC
PHOX2B_R	CTGGTGAAAGTGGTGCGGAT
HAND1_1F	TCCGCAGAAGGGTTAAACAGG
HAND1_1R	AGCCCACTGTCTTCTTACCG
HAND2_F	AAGGACGACCAGAATGGCG
HAND2_R	ATTTCGTTCAGCTCCTTCTTCC
GATA3_1F	CTCTTCGCTACCCAGGTGAC
GATA3_1R	ACGACTCTGCAATTCTGCGA
TBX2_F	GGCCTTCCACAAGCTGAAG
TBX2_R	GCGGCTGGTACTTGTGCAT
ISL1_F	GGCATGTTTGAAATGTGCGG
ISL1_R	ACACAGCGGAAACACTCGAT
NTRK1_F	ATCTTCACCTACGGCAAGCA
NTRK1_R	TAGCCCAGGACATCCAGGTA
DPYSL3_F	GGACAACTTCACAGCCATTCCTG
DPYSL3_R	GTGCTTGTCACAGCCACGAACT
TUBB3_F	TCAGCGTCTACTACAACGAGGC
TUBB3-R	GCCTGAAGAGATGTCCAAAGGC
HPRT_F	TGACACTGGCAAAACAATGCA
HPRT_R	GGTCCTTTTCACCAGCAAGCT

Suppl. Table 5: Antibodies

1°Antibody	Company	Dilution
MYCN	Abcam (ab16898)	1:1000
TBX3	Santa Cruz (sc-16623)	1:500
ETS1	Santa Cruz (sc-55581)	1:500
HIF1A	BD Pharmigen (610958)	1:500
GATA3	Santa Cruz (sc-9009 X)	1:1000
GATA2	Santa Cruz (sc-9008X)	1:2000
SOX11	Abcam (ab170916)	1:200
SOX4	Santa Cruz (sc-518016)	1:500
HEY1	Abcam (ab154077)	1:500
ID2	Santa Cruz (sc-398104)	1:500
TWIST1	Santa Cruz (sc-6269)	1:500
SMAD9	Abcam (ab96698)	1:1000
PHOX2A	Abcam (ab168392)	1:1000
GAPDH	Santa Cruz (sc-25778)	1:2000

Suppl. Table 6: ChIP –qPCR primers

Primer Name	Sequence (5'-3')
ChIP_SOX11_3F	CTGGAAGAAAAAGGTATTCAGCA
ChIP_SOX11_3R	TTGATGGAGTCTGGGTGACA
ChIP_SOX11_4F	GCATTTTGAATCTGGATGCTG
ChIP_SOX11_4R	TGTTTGTGAAGCAGAAAAACATT
ChIP_SOX11_5F	TGTTTTTCATTCTAATGGCTGTTT
ChIP_SOX11_5R	GCTAGCAGGAAGCAGTAGCA
ChIP_SOX4_3F	TTCAAAAATGATGTGTTTCACTG
ChIP_SOX4_3R	CCTTCAAGCCGGATGTACTTT
ChIP_SOX4_4F	CCTAAGCCCCTAAATACAAGAATG
ChIP_SOX4_4R	GCAACAAACACGAAACTCCA
ChIP_SOX4_5F	AATTGAAAAGAGCTGGCCATT
ChIP_SOX4_5R	GGCTACAGGAACCATGAAGTT
LMO1_Primer 1_F	GGCTGGTCCAGGAGACTTGA
LMO1_Primer1_R	GGACCCTGTGGGGAGCTTAT
LMO1_Primer 2_F	AGCATCCCTTTGCTGAAGTCC
LMO1_Primer2_R	CCTTTCCTGAAGGAGCGCAA
GAPDH Primer1	ATGGTTGCCACTGGGGATCT
GAPDH Primer 2	TGCCAAAGCCTAGGGGAAGA

Source Data: Uncropped Western blots of Supplementary Figures

Supplementary Figure 1f:

H3K27ac:

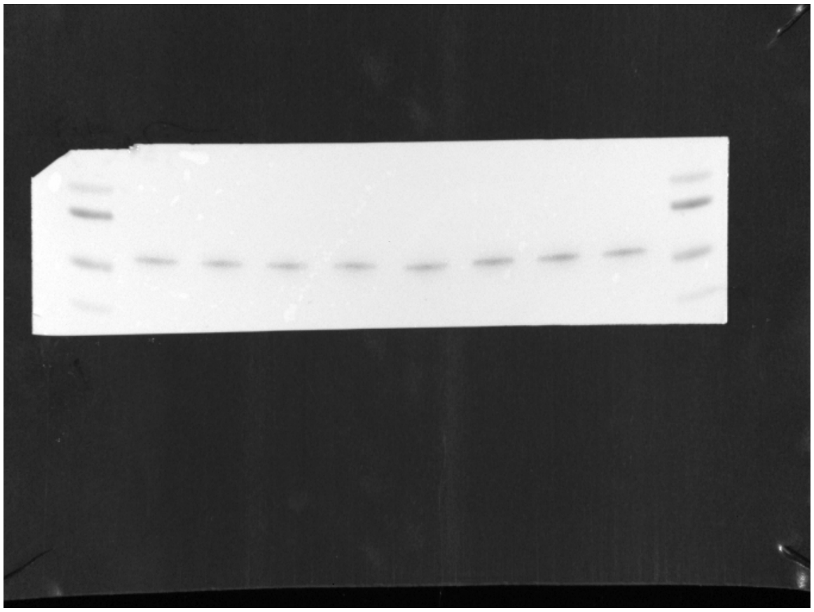

Histone H3:

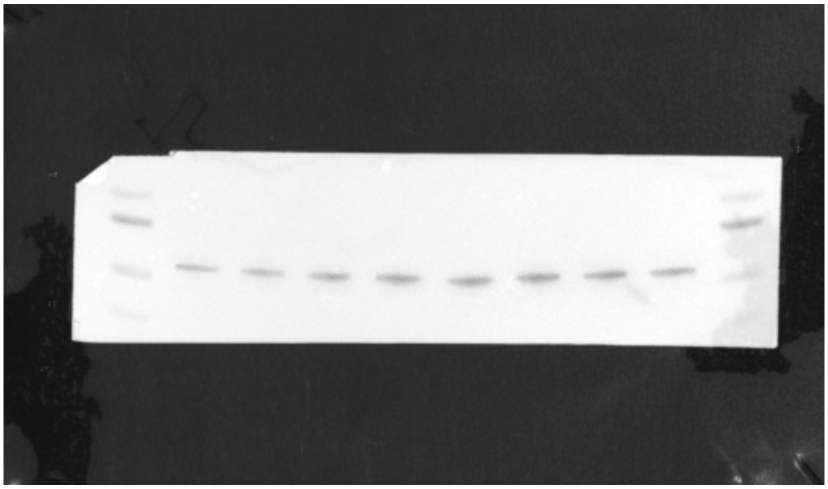

Uncropped western blots from Supplementary Fig 3f:

ETS1:

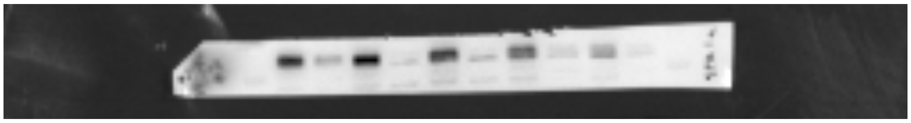

GAPDH:

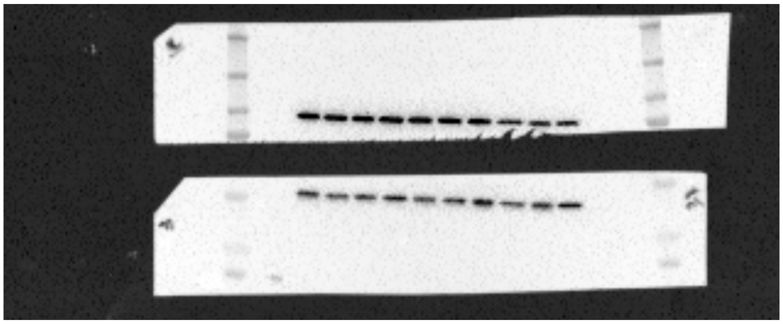

TBX2:

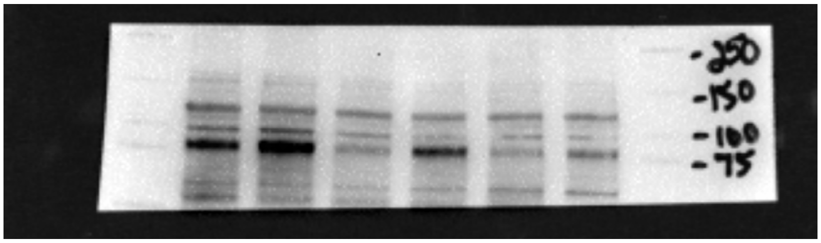

MYCN:

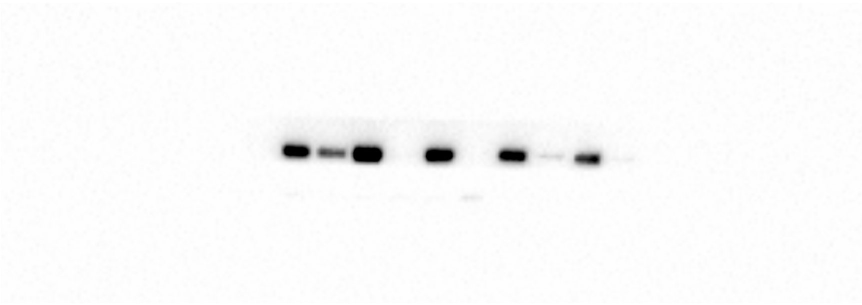

PHOX2A:

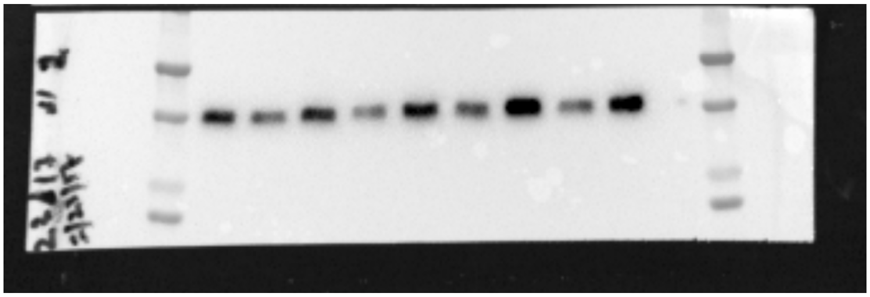

SMAD9:

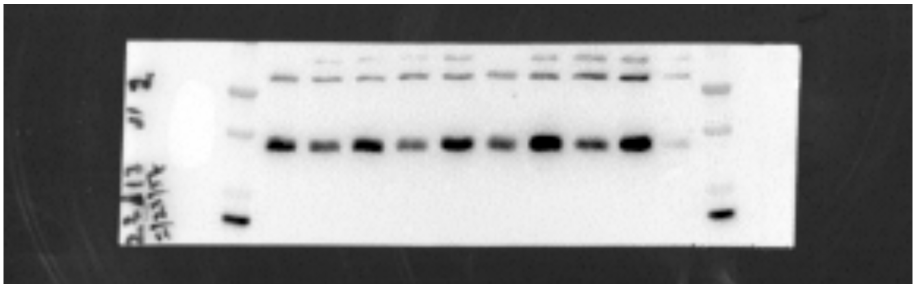

ID2:

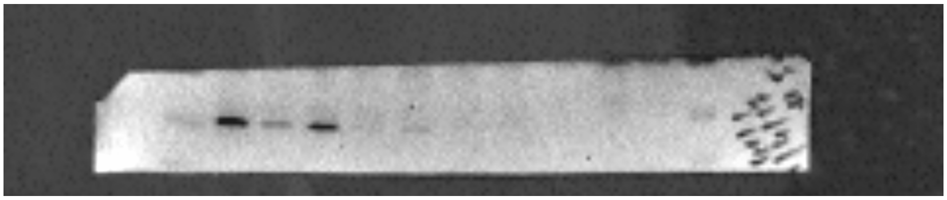

HEY1:

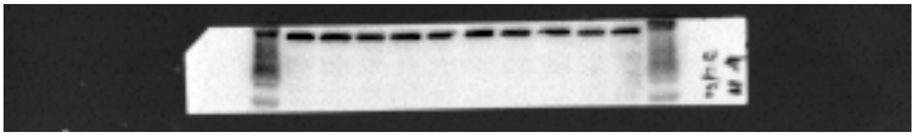

GATA2:

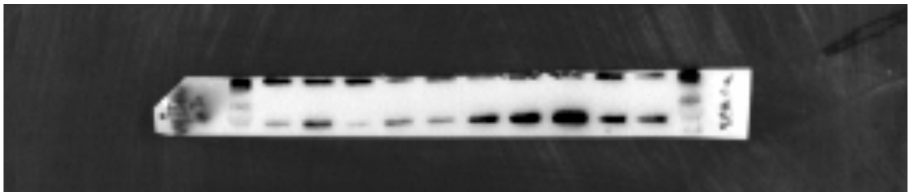

SOX4:

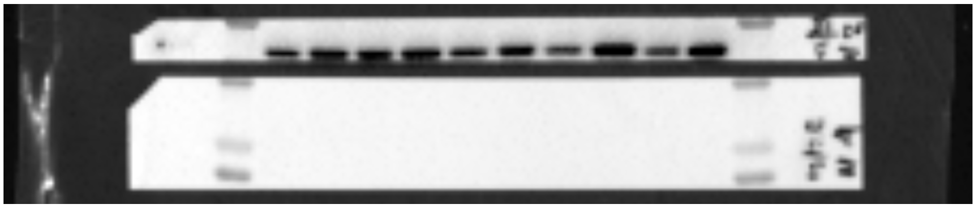

SOX11:

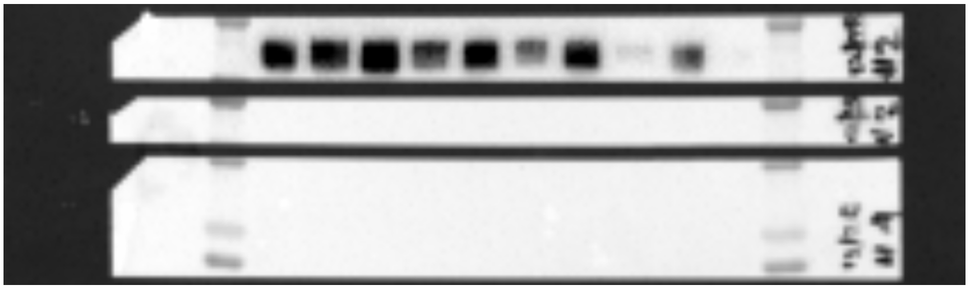

Supplement: Supplementary file 1 — Supplementary Figs. [file 41467_2024_47166_MOESM1_ESM.pdf]
