# Supplementary material for: Lineage specific transcription factor waves reprogram neuroblastoma from self-renewal to differentiation
Source: Nat Commun. 2024 Apr 23;15:3432. doi: 10.1038/s41467-024-47166-y (PMC11039666; doi:10.1038/s41467-024-47166-y)
Supplement: Supplementary file 3 — Description of Additional Supplementary Files [file 41467_2024_47166_MOESM3_ESM.pdf]

<u>Description of Additional Supplementary Files</u>

Supplementary Dataset 1:

Temporal patterns of SE changes and identified 1199 SEs in KCNR cells under self-renewal and differentiating conditions. Signal is reported as the reads per million of H3K27ac ChIP-seq signal.

Supplementary Dataset 2:

Overlap of GWAS SNPs with super enhancers (tab 1), all neuroblastoma SNPs reported in McDaniel, L. D. *et al.* Common variants upstream of MLF1 at 3q25 and within CPZ at 4p16 associated with neuroblastoma. *PLoS genetics* **13**, e1006787 (2017), tab 2. Tab 3 contains the bed coordinates for all super enhancers in KCNR cells used for the overlap with GWAS coordinates.

Supplementary Dataset 3:

Each tab is for a different super enhancer cluster (Lost, 1st wave, 2nd wave and 3rd wave). Data shown are for H3K27ac ChIP-seq signal over a time course of ATRA treatment, alongside average gene expression levels over the same ATRA treatment from RNA-seq of SE-associated genes. The Pearson Correlation is additionally calculated between SE signal and gene expression.

Supplementary Dataset 4:

HOMER motif analysis results for transcription factor motifs enriched in SOX4 CUT&RUN peaks. Known motifs are reported, alongside statistical significance and the number and percentages of target sequences with the motif, and the occurrence of the motif in background sequences.

Supplementary Dataset 5:

The coordinates of SOX4 peaks increased upon ATRA treatment (1st tab), and a list of associated genes increased near SOX4 gained peaks (2nd tab).

Supplementary Dataset 6:

List of genes near SOX11 binding sites that decreased in expression after ATRA treatment in both KCNR and LAN5 cells.

Supplementary Dataset 7:

Individual neuroblastoma cell line data for the SOX11 Gene Effect (Chronos) CRISPR (DepMap 21Q3) and the gene expression of *SOX11* log2(TPM+1) from the DepMap project. MYCN amplification status is added for each cell line.

Supplementary Dataset 8:

Ingenuity Pathway Analysis of siSOX11 RNA seq data, indicating categories and their significance, and a list of the genes in each pathway, and the predicted activation.

Supplementary Dataset 9:

A list of the number of cell line tumor types in the DepMap analysis, related to Figure 7a.
