# Supplementary material for: Lineage specific transcription factor waves reprogram neuroblastoma from self-renewal to differentiation
Source: Nat Commun. 2024 Apr 23;15:3432. doi: 10.1038/s41467-024-47166-y (PMC11039666; doi:10.1038/s41467-024-47166-y)
Supplement: Supplementary file 13 — Reporting Summary [file 41467_2024_47166_MOESM13_ESM.pdf]

nature research

Corresponding author(s): Deblina Banerjee, Berkley E. Gryder, Carol J Thiele

Last updated by author(s): Mar 4, 2024

Reporting Summary

Nature Research wishes to improve the reproducibility of the work that we publish. This form provides structure for consistency and transparency in reporting. For further information on Nature Research policies, see our Editorial Policies and the Editorial Policy Checklist.

Statistics

For all statistical analyses, confirm that the following items are present in the figure legend, table legend, main text, or Methods section.

n/a	Confirmed	
☐	☒	The exact sample size (*n*) for each experimental group/condition, given as a discrete number and unit of measurement
☐	☒	A statement on whether measurements were taken from distinct samples or whether the same sample was measured repeatedly
☐	☒	The statistical test(s) used AND whether they are one- or two-sided<br>*Only common tests should be described solely by name; describe more complex techniques in the Methods section.*
☐	☒	A description of all covariates tested
☐	☒	A description of any assumptions or corrections, such as tests of normality and adjustment for multiple comparisons
☐	☒	A full description of the statistical parameters including central tendency (e.g. means) or other basic estimates (e.g. regression coefficient) AND variation (e.g. standard deviation) or associated estimates of uncertainty (e.g. confidence intervals)
☐	☒	For null hypothesis testing, the test statistic (e.g. *F*, *t*, *r*) with confidence intervals, effect sizes, degrees of freedom and *P* value noted<br>*Give P values as exact values whenever suitable.*
☒	☐	For Bayesian analysis, information on the choice of priors and Markov chain Monte Carlo settings
☒	☐	For hierarchical and complex designs, identification of the appropriate level for tests and full reporting of outcomes
☐	☒	Estimates of effect sizes (e.g. Cohen's *d*, Pearson's *r*), indicating how they were calculated

Our web collection on statistics for biologists contains articles on many of the points above.

Software and code

Policy information about availability of computer code

Data collection

Sequencing data collection was performed with Illumina's NextSeq 550 system. Code used to analyze the data is all publicly available here: https://github.com/CBIIT/ChIP_seq

Data analysis

Software and Algorithms
HiC-pro (v2.11.1) https://github.com/nservant/HiC-Pro
Juicebox (v1.11.08) https://github.com/aidenlab/Juicebox
Juicer (v1.5.6) https://github.com/aidenlab/juicer
STAR (v2.5.3a) https://github.com/alexdobin/STAR
RSEM (v1.3.2) https://github.com/deweylab/RSEM
MSIM package (v1p0) https://code.google.com/archive/p/msim/source
R statistical package drc Christian Ritz https://cran.r-project.org/web/packages/drc/drc.pdf
Seurat (v2.3.0) Satija Lab https://github.com/satijalab/seurat/
Drop-seq software tools (v.1.2) McCarroll lab http://mccarrolllab.com/wp-content/uploads/2016/03/Drop-seqAlignmentCookbookv1.2Jan2016.pdf)
NGSplot (v. 2.63) Shen Lab https://github.com/shenlab-sinai/ngsplot
MACS (v 2.1.1.20160309) Zhang et al 2008 https://github.com/taoliu/MACS
BWA (v 0.7.17) Li and Durbin, 2009 http://bio-bwa.sourceforge.net/
HOMER (Hypergeometric Optimization of Motif EnRichment) version 4.9.1 Heinz et al, 2010 http://homer.ucsd.edu/homer/index.html
ImageJ NIH https://imagej.nih.gov/ij/
Graphpad Prism (v7.01) Graphpad Software https://www.graphpad.com/scientific-software/prism/
ROSE2 Charles Lin Lab https://github.com/linlabbcm/rose2
Coltron Lin et al, 2016 https://pypi.org/project/coltron/
Bamliquidator version 1.3.4 John DiMatteo https://github.com/BradnerLab/pipeline/wiki/bamliquidator
GSEA software version 2.2.0 Subramanian et al, 2005 https://software.broadinstitute.org/gsea/

Bedtools version 2.27.1 Quinlan and Hall, 2010 http://bedtools.readthedocs.io/en/latest/
RNA-seq Plotting R Scripts This paper https://github.com/GryderArt/VisualizeRNAseq
Deseq2 https://github.com/mikelove/DESeq2

For manuscripts utilizing custom algorithms or software that are central to the research but not yet described in published literature, software must be made available to editors and reviewers. We strongly encourage code deposition in a community repository (e.g. GitHub). See the Nature Research guidelines for submitting code & software for further information.

Data

Policy information about availability of data

All manuscripts must include a data availability statement. This statement should provide the following information, where applicable:

- Accession codes, unique identifiers, or web links for publicly available datasets
- A list of figures that have associated raw data
- A description of any restrictions on data availability

The raw sequencing data and processed files were deposited in the GEO repository at the NCBI (GEO: GSE147408).

Field-specific reporting

Please select the one below that is the best fit for your research. If you are not sure, read the appropriate sections before making your selection.

☒ Life sciences ☐ Behavioural & social sciences ☐ Ecological, evolutionary & environmental sciences

For a reference copy of the document with all sections, see nature.com/documents/nr-reporting-summary-flat.pdf

Life sciences study design

All studies must disclose on these points even when the disclosure is negative.

Sample size	No sample size calculations were performed.
Data exclusions	Regions of the genome on the ENCODE blacklist (comprised of, for instance, highly repetitive regions) were excluded from called peaks prior to all downstream analysis (ie, prior to super enhancer calling in the case of H3K27ac ChIP-seq data)
Replication	All experiments were performed in at least duplicate and often triplicate (biologically independent), where possible over multiple cell lines, and strengthened by taking genome wide measurements over a time course series.
Randomization	No samples were needed to be randomized.
Blinding	No blinding was necessary or carried out for any of the experiments described herein.

Reporting for specific materials, systems and methods

We require information from authors about some types of materials, experimental systems and methods used in many studies. Here, indicate whether each material, system or method listed is relevant to your study. If you are not sure if a list item applies to your research, read the appropriate section before selecting a response.

Materials & experimental systems

n/a	Involved in the study	
☐	☒	Antibodies
☐	☒	Eukaryotic cell lines
☒	☐	Palaeontology and archaeology
☒	☐	Animals and other organisms
☒	☐	Human research participants
☒	☐	Clinical data
☒	☐	Dual use research of concern

Methods

n/a	Involved in the study	
☐	☒	ChIP-seq
☒	☐	Flow cytometry
☒	☐	MRI-based neuroimaging

Antibodies

Antibodies used	antibodies targeting H3K27ac (Active Motif, catalog #39133), H3K4me3 (Cell Signaling, catalog # 9751), H3K4me1 (Abcam, catalog # ab8895), and H3K9me3 (Active Motif, catalog # ab8898)
Validation	antibodies were validated by the companies that sold them.

Eukaryotic cell lines

Policy information about cell lines

Cell line source(s)	KCNR, LAN5: COG (https://www.cccells.org/cellreqs-nbl.php),<br>IMR32: ATCC (https://www.atcc.org/products/all/CCL-127.aspx)<br>SY5Y: ATCC (https://www.atcc.org/products/all/CRL-2266.aspx)<br>Kelly: Gift from Kim Stegmaier, DSMZ (https://www.dsmz.de/collection/catalogue/details/culture/ACC-355)<br>NGP: Gift from Garrett Brodeur, DSMZ (https://www.dsmz.de/collection/catalogue/details/culture/ACC-676)
Authentication	STR Profiling
Mycoplasma contamination	All cell lines were negative for mycoplasma contamination
Commonly misidentified lines<br>(See ICLAC register)	NA

ChIP-seq

Data deposition

☒ Confirm that both raw and final processed data have been deposited in a public database such as GEO.

☒ Confirm that you have deposited or provided access to graph files (e.g. BED files) for the called peaks.

Data access links<br>*May remain private before publication.*	https://www.ncbi.nlm.nih.gov/geo/query/acc.cgi?acc=GSE147408<br>reviewer token: wnyxwaosvnwrlwx
Files in database submission	*Provide a list of all files available in the database submission.*
Genome browser session<br>(e.g. UCSC)	*Provide a link to an anonymized genome browser session for "Initial submission" and "Revised version" documents only, to enable peer review. Write "no longer applicable" for "Final submission" documents.*

Methodology

Replicates	Replicates were performed for each condition (2, 4, and 8 days), and repeated independently across 2 separate cell lines (LAN5 and KCNR)
Sequencing depth	Sequencing was performed to a depth of more than 40 million reads per sample/condition/cell line.
Antibodies	antibodies targeting H3K27ac (Active Motif, catalog #39133), H3K4me3 (Cell Signaling, catalog # 9751), H3K4me1 (Abcam, catalog # ab8895), and H3K9me3 (Active Motif, catalog # ab8898)
Peak calling parameters	ChIP-Seq peaks were detected using MACS2 (version 2.1.0) 64. The default p value cut off for peak enrichment was set to 10-5 for all data sets.
Data quality	*Describe the methods used to ensure data quality in full detail, including how many peaks are at FDR 5% and above 5-fold enrichment.*
Software	Code used to analyze the data is all publicly available here: https://github.com/CBIIT/ChIP_seq
